# The evolution of similarity-biased social learning

**DOI:** 10.1017/ehs.2024.46

**Published:** 2025-01-20

**Authors:** Paul E. Smaldino, Alejandro Pérez Velilla

**Affiliations:** 1 Department of Cognitive & Information Sciences, University of California, Merced, Merced, CA, USA; 2 Santa Fe Institute, Santa Fe, NM, USA

**Keywords:** cultural evolution, social identity, social identity, social learning strategies, parochialism, diversity

## Abstract

Humans often learn preferentially from ingroup members who share a social identity affiliation, while ignoring or rejecting information when it comes from someone perceived to be from an outgroup. This sort of bias has well-known negative consequences – exacerbating cultural divides, polarization, and conflict – while reducing the information available to learners. Why does it persist? Using evolutionary simulations, we demonstrate that similarity-biased social learning (also called parochial social learning) is adaptive when (1) individual learning is error-prone and (2) sufficient diversity inhibits the efficacy of social learning that ignores identity signals, as long as (3) those signals are sufficiently reliable indicators of adaptive behaviour. We further show that our results are robust to considerations of other social learning strategies, focusing on conformist and pay-off-biased transmission. We conclude by discussing the consequences of our analyses for understanding diversity in the modern world.

## Graphical abstract

**Figure:**
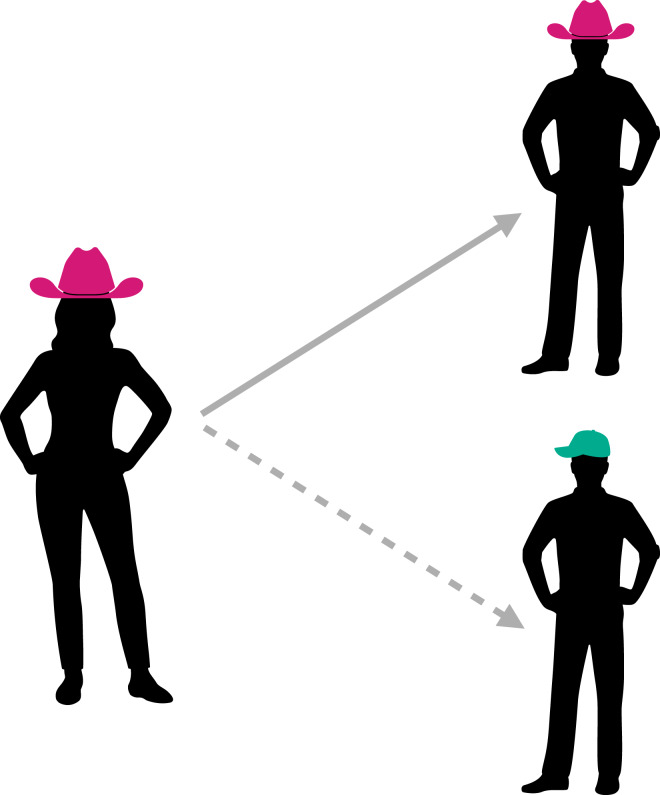

## Social media summary

Why do we often prefer to learn from people who are similar to us and ignore people we view as different? We present a cultural evolutionary model that demonstrates how, in diverse populations, parochial social learning can be adaptive whereas other social learning strategies – like learning from successful individuals or averaging over many observations – can fail. We discuss implications for stereotypes and polarization.

## 1. Introduction

Humans are obligate social learners (Boyd, 2018; Boyd et al., 2011; Henrich, 2015). But we don’t learn from just anyone. Instead, we often learn preferentially from ingroup members who share a social identity affiliation, while we may ignore or reject information or advice when it comes from someone perceived to be from an outgroup (Burke & Stets, 2022; Chudek et al., 2013; Cruz y Celis Peniche & Moya, 2024; Ehret et al., 2022; Moya, 2023; Smaldino et al., 2017). That is, humans often practice similarity-biased social learning, which we can also call *parochial social learning*. This sort of bias has some well-known negative consequences; helping to exacerbate cultural divides, polarization, and intergroup conflict (DellaPosta et al., 2015; Durham, 1991; Richerson et al., 2016). It also entails ignoring information that might be useful, even disregarding interventions that would counter existential threat (Smaldino & Jones, 2021).

Why do we act like this? In this paper, we present a functional explanation for the phenomenon of parochial social learning and explore the conditions for its adaptive evolution. We propose that in diverse environments, parochial social learning helps direct social learning toward targets most likely to have information relevant to the learner. We will demonstrate the plausibility of this proposal with computational modeling, and we will further show that the proposal is robust even when a parochial bias interacts with other social learning strategies such as conformist or pay-off-biased transmission.

Cultural evolutionary theory has focused extensively on social learning, and with good reason. Dual inheritance implies two channels for the transmission of traits: genes and culture, the latter of which operates largely (if not entirely) through learning from others. Theoretical models have shown why individuals are expected to rely more on social learning in cases where individual learning can’t be trusted – for example, in noisy, complex environments with many options (Aoki & Feldman, 2014; Boyd & Richerson, 1985; McElreath et al., 2013; Turner et al., 2023). For social learning to be adaptive, individuals must have information that others can use and that would be easier to acquire through social transmission than by costly or risky individual trial and error. However, if the world is *so* noisy that what has worked for others is unlikely to work for us, social transmission may also fail to provide learners with adaptive traits. Thus, the evolution of social learning requires at least some spatiotemporal autocorrelation in adaptive behaviour (Aoki & Feldman, 2014; Boyd & Richerson, 1985; McElreath et al., 2013).

Theories of social learning, bolstered by formal modelling, have identified several strategies that can boost the efficacy of social learning and mitigate some sources of uncertainty, so that social learning is adaptive under a wider range of conditions. For example, conformist transmission (also called consensus learning) reduces noise by giving preferential treatment to behaviours adopted by a majority, giving minimal weight to rare behaviours (even when they might be innovative) (Perreault et al., 2012; Whiten, 2019). Pay-off-biased transmission (also called success bias) gives preferential weight to behaviours adopted by individuals who exhibit visible signs of success, with the presumption (or at least the hope) that behaviours employed by successful individuals are likely to be causally related to their success (Baldini, 2013). These strategies outperform unbiased social learning, in which targets are copied at random, under a wide range of conditions (but see McElreath et al. (2013) and Baldini (2013) for exceptions).

Much of the theoretical literature on social learning strategies has assumed that individuals live in relatively homogeneous groups, and that behaviours that lead to success for one person will lead to success for others in the same environment. This assumption may sometimes be valid. But it is not *always* valid, particularly in diverse, multi-ethnic, stratified, or cosmopolitan societies. In these societies, many types of people can live side by side yet experience very different opportunities and affordances, economic as well as social. Evaluating the nature and value of social information in such diverse environments is likely to be challenging. Indeed, humans living in modern industrialized societies are regularly exposed to a dizzying number of sources, each providing varied and often divergent information.

Many cognitive and cultural features are mechanisms for parsing and organizing information in the world to reduce uncertainty. Categorizing situations and events into broad types and schemas helps us to predict what is likely to happen in those situations and what decisions we will likely need to make (Barsalou, 2021; Markman & Ross, 2003; Smaldino & Richerson, 2012). Categories also aid communication, as we can use shorthand to describe situations, actions, objects, and people to others. Social categories are no different. We readily parse other people into categories that help us to predict their likely behaviour, that prepare the scope of our interactions with them, and help us describe them to others (Smaldino, 2019).

Markers of social identity provide utility in identifying targets for likely cooperation or conflict (Barth, 1969; Boyd & Richerson, 1987; Efferson et al., 2008; McElreath et al., 2003; Moffett, 2019; Moya, 2023; Smaldino, 2019). Such markers can also help humans identify targets for social learning. Gender, for example, might be a universal human filter for social learning (Chudek et al., 2013; Cruz y Celis Peniche & Moya, 2024), because so many behaviours in such a wide range of societies cluster by gender. In diverse populations, ethnic or otherwise disadvantaged minorities and those with lower socioeconomic class markers may not have the social capital or clout to successfully perform behaviours that members of dominant majority groups or elite socioeconomic classes can get away with (Bourdieu, 1986; Bunce, 2021). For example, in some parts of the United States, risk-taking behaviours – such as confidently questioning authority figures – may help a wealthy white man land a job or get out of a speeding ticket, whereas the same behaviours performed by a poor minority individual could lead to charges of insubordination, arrest, or worse (de Courson et al., 2024; Schuck et al., 2008). When disparities like this exist, people might be better off learning behaviours from individuals whose circumstances better match their own, and indeed such preferential learning from the ingroup is often encouraged within many subpopulations of all genders and social classes (Bowles & Gintis, 2004).

In the modern world, mass media, social media, and the Internet exacerbate the need to use simplifying heuristics to categorize information sources, because we are exposed to vast amounts of social information from all over the world, often divorced from the context that would help us parse its meaning and value (Donath, 2020). Different groups of people have different associations with particular words or concepts, and when those words or concepts are invoked across contexts, disagreement can arise. In today’s polarized United States, political identity has become a beacon for differentiation (Mason, 2018). For example, Americans on the political left and right have very different ideas of what is implied by the label ‘racist’, with people on the right denoting as racist the social movement #BlackLivesMatter and people on the left similarly finding the countermovement #AllLivesMatter particularly racist (Powell et al., 2023). Similarly, when asked to name ‘socialist’ countries, the top three answers given by Republican voters in the United States were Venezuela, China, and Russia, whereas the top three answers given by Democratic voters were Denmark, Sweden, and Norway (Smith, 2020). Divergent usage of the same word limits the ability of individuals to productively engage with and learn from others even within the same society. In a recent study, Kim et al. (2023) found that having Twitter posts marked as misinformation led posters to decrease rather than increase the political diversity of the content they engaged with online, having apparently learned that engaging with political outgroups, even in good faith, leaves them subject to online attacks. And authority figures on either side of the political spectrum regularly take efforts to paint the other side as untrustworthy (Alyukov, 2023). Such phenomena may increase the extent to which individuals in the modern world are incentivized to rely on parochial social learning.

What about other approaches that can boost the signal of adaptive social information: strategies like conformist or pay-off-biased transmission? Defining these strategies is not so straightforward in diverse societies. It is unclear whether a conformist learner should copy the majority if their own group is not well-represented by that majority. Groups may likewise disagree about the criteria by which someone is judged successful or prestigious. A person deemed worthy of esteem and emulation in one group may be the subject of scorn, mockery, or indifference in another.

Prior modelling work has shown that selection for social learning decreases when it becomes more likely that learners and targets are from different environments with different adaptive behaviours (Aoki & Feldman, 2014; Boyd & Richerson, 1985; McElreath et al., 2013). Here, we build on this idea and propose a workaround that enables the selective targeting of individuals with similar adaptive circumstances: signals of social identity. If people can take advantage of markers or cues of identity to preferentially learn from targets whose behaviours are likely to also work for themselves, they can boost the efficiency of social learning. A bias for similar targets may allow social learning to evolve in situations where unbiased learning – or even learning bolstered by conformist or pay-off-biased proclivities – would not otherwise be favoured.

There is ample evidence that people do in fact have preferences for learning for similar others (Chudek et al., 2013; Cruz y Celis Peniche & Moya, 2024; Moya, 2023; Rosekrans, 1967; Wood et al., 2013). Children typically prefer to learn from individuals who match their gender identity (Perry et al., 2019; Shutts et al., 2010; Wolf, 1973), and often prefer to learn from individuals with similar accents and speech patterns starting at very young ages (Kinzler, 2021), later developing similar (if perhaps weaker) biases for learning from those who share racial identities (Kinzler et al., 2009). The appropriateness of some norms and behaviours are age-specific, and both children and adults reliably copy normative behaviour (such as styles and dress) from age-peers, particularly in instances when consideration of coordination or conformity weigh heavily. That said, peer-learning appears to be particularly common in modern, Western societies, whereas elsewhere age cues are often discounted in favour of other signals of similarity, seniority, or prestige (Kline et al., 2013; Mesoudi et al., 2016; Reyes-García et al., 2016). In diverse populations, cultural biases may also restrict learners from adopting products or behaviours associated with outgroups, a phenomenon sometimes called ‘outgroup aversion’ (Berger & Heath, 2008; Ehret et al., 2022; Smaldino et al., 2017; Smaldino & Jones, 2021).

Of course, people can be similar or different in many ways, and identity is multidimensional and complex (Golder & Donath, 2004; Roccas & Brewer, 2002; Smaldino, 2019; Wolpert et al., 2011). Dimensions of identity include ethnicity, culture, gender, sexual orientation, age, socioeconomic status, social capital, cultural and linguistic heritage, even sports fandom. The adaptive value of learning preferentially from similar others will depend on the extent to which context-specific adaptive behaviours correlate with particular identity aspects (Witt et al., 2023; Wood et al., 2013). When identity is not a reliable indicator of adaptive information, we should not expect social learning to attune to it.

The adaptive advantage of learning from similarly marked others has been proposed by numerous authors (Boyd & Richerson, 1987; Henrich, 2015; McElreath et al., 2003; Montrey & Shultz, 2022; Moya, 2023; Wood et al., 2013). Given the preponderance of evidence that people do in fact have such biases, it seems important to formalize the claim and explore both its coherence and its potential limits. Yet the adaptive value of parochial social learning has not been explicitly considered using formal models. One exception is a recent study by Saunders (2023). Using a modelling framework very different from ours, Saunders claimed that similarity-biased learning was not favoured under most scenarios, or at least that it did not improve performance. However, this study focused only on pay-off-biased social learning in a coordination game in which there was both strong between-group differences in norms and strong homophilic assortment for interactions. Saunders found that pay-off-biased copying was often sufficient to achieve optimal pay-off outcomes without the need for similarity-biased learning. Critically, however, group salience was already present, such that two groups with divergent norms needed to solve the problem of assortment but could use miscoordination as feedback to update their behaviours. In other words, because Saunder’s model assumes the pre-existence of identity-based homophily in game play, it negates the need for ‘similarity-biased learning’ only because players get pay-off feedback (another form of learning) from a similarity-biased sample of co-players. Moreover, because the only learned trait in this model was related to coordination, a pay-off-maximizing outcome was for all agents to simply adopt the same trait value, negating any deeper group differences. In contrast, we consider a more general model of social learning in which the only social interaction is the imitation of environmentally adaptive behaviours, more in line with the broader literature on the evolution of social learning.

In the following sections, we will describe a formal model for the evolution of similarity-biased social learning, focusing on learning a continuously variable behaviour in an environment in which individual learning is error-prone and in which different individuals may benefit maximally from adopting different behaviours. We will first show that unbiased social learning evolves when individual learning is more error-prone and that the adaptive value of social learning relies on adaptive behaviours being correlated between learners and targets, in line with prior research. We will then show that a parochial bias can stabilize the adaptive value of social learning even when adaptive behaviours vary among individuals, as long as identity markers correlate with adaptive behaviours. Finally, we show that our results are robust to considerations of other social learning strategies, focusing on conformist and pay-off-biased transmission. Although both strategies are typically bolstered by parochialism in diverse populations, we find that pay-off-biased transmission can potentially impede the benefits of parochialism from being realized in disadvantaged groups, particularly when identity signals are not fully reliable indicators of adaptive behaviours.

## 2. Model

Consider a population of 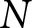 individuals divided into two intermixed groups (representing differentiation on norms, affordances, etc. in a cosmopolitan society). The proportion of the population in group 0 is 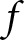, with the remaining 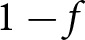 in group 1. Each individual 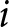 is characterized by five heritable traits: their type (or group) 
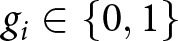
, their marker 
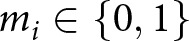
, their parochialism 
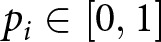
, their reliance on social learning 
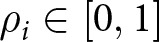
, and their social learning strategy, 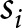 (unbiased, conformist, or pay-off-biased transmission).

**Figure 1.**
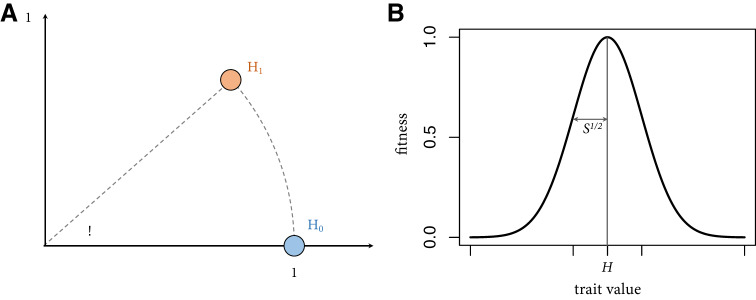
(A) Depiction of the two adaptive traits, in polar coordinates. 
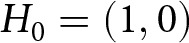
, 
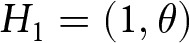
. (B) Gaussian fitness function. The horizontal axis represents the distance of the agent’s trait value from their group-specific optimum, 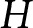, and the vertical axis is the corresponding pay-off.

During their lifespan, each individual 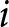 acquires a behavioural trait 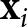 using one of several learning strategies. We represent traits as points in continuous two-dimensional space, which is a minimal representation of the idea that traits have multiple components, and also helps us avoid path-dependent effects possible with a one-dimensional representation in which being closer to one adaptive value necessitates being further from another. For each group 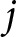, there is an optimally adaptive trait value, 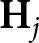, which maximizes the fitness an individual of that group can receive. Without loss of generality, we set these adaptive trait values on the unit circle; we can represent their positions in polar coordinates as 
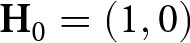
 and 
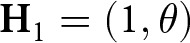
 (see Figure 1A). We restrict 
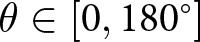
, representing the spectrum from aligned to orthogonal to opposed trait values between the two groups.

Individuals can acquire trait values through both individual and social learning. Social learning is presumed to be accurate and costless. Individual learning, however, is less reliable, and we follow Boyd and Richerson (1985) in modelling the costs of individual learning as the risk of adopting a suboptimal trait value. Below we describe how the model is initialized followed by a detailed description of the model dynamics. See Tables 1 and 2 for a list of model parameters.

**Table 1.** Global model parameters

Parameter	Meaning	Value(s)
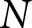	Population size	{50, 200}
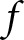	Proportion of the population in the larger group	[0.5, 1]
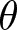	Distance between the two adaptive traits	[ 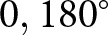 ]
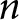	Number of models for social learning	{1, 5, 15}
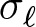	Individual learning uncertainty	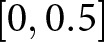
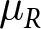	Mutation rate for social learning reliance	{0, 0.05}
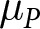	Mutation rate for parochialism	{0, 0.05}
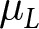	Mutation rate for learning strategy	{0, 0.01}
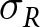	Standard deviation of social learning reliance mutation	0.05
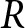	Group marker reliability	[0, 1]
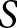	Intensity of selection	0.05

**Table 2.** Agent traits

Trait	Meaning	Values
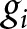	Agent type/group	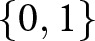
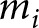	Agent marker	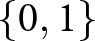
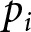	Parochialism	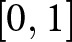
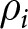	Reliance on social learning	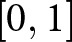
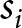	Learning strategy	{UT, CT, PT}

### 2.1 Initialization

At initialization, agents are created and assigned to one of the two type-groups. Specifically, an agent is assigned to group 0 with probability 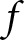 and group 1 otherwise. Agents are also assigned markers, which may or may not be reliable indicators of their group (that is, the adaptive trait that works for them). With probability 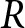, agents are assigned the group marker that corresponds to their group, so that 
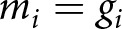
. Otherwise, agents are assigned a marker with probability equal to the frequency of the associated adaptive trait (i.e. they are assigned marker 0 with probability 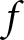 and marker 1 with probability 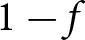), so as to keep the relative frequency of markers consistent across conditions. This allows for a direct comparison between runs with different values of 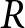 for a given value of 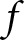. 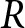 represents the extent to which markers are reliable indicators of group identity. When 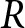 is close to 1, the markers are strongly correlated with the adaptive trait values of the agents who use them; when 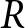 is close to zero, they are not very informative. We assume that all agents begin as non-parochial individual learners, so that 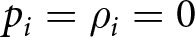 at initialization.

The model dynamics consist of discrete, non-overlapping generations that may learn socially from members of the previous generation. Because the first generation relies entirely on individual learning, each agent 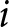 initially adopts a trait value equal to 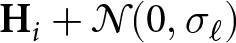, such that their learned trait values are points in Cartesian space drawn from a bivariate normal distribution centred on 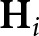 and a normalized standard deviation of 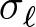. Each agent then acquires a pay-off based on their trait value, described below.

### 2.2 Dynamics

After initialization, the model proceeds in discrete time steps, which are broken up into four stages: (1) reproduction, (2) model choice, (3) learning, and (4) pay-off acquisition.

#### 2.2.1 Reproduction

A new generation of 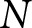 individuals is created. Each group produces a number of offspring equal to its current size (to keep both the population size and the relative group sizes constant). Each agent in the new generation has one parent, chosen from its own group with probability equal to the relative pay-off of the parents in that group. Specifically, an agent 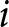 in group 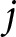 in the parent generation is selected at random, and reproduces with a probability
(1)
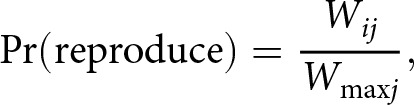


where 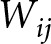 is the agent’s pay-off and 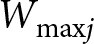 is the highest pay-off in the group among the parent generation. In this way, learning strategies associated with higher pay-offs will proliferate.

Each offspring inherits the social learning strategies and parochialism of its parent, although imperfectly. With probability 
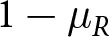
, the offspring inherits the social learning reliance of its parent. Otherwise the agent 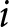 sets its social learning reliance to 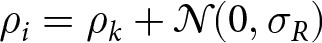, where 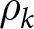 is the social learning reliance of the parent, and the mutation amount is a randomly drawn value from a normal distribution with a mean of zero and a standard deviation of 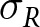. The value of 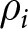 will be truncated as necessary to remain in the range [0, 1]. Parochialism is transmitted in the same way. With probability 
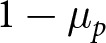
, the offspring inherits the parochialism of its parent. Otherwise the agent 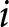 sets its parochialism to 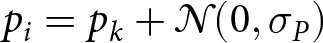, where 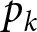 is the parochialism of the parent, and the mutation amount is a randomly drawn value from a normal distribution with a mean of zero and a standard deviation of 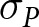. The value of 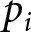 will be truncated as necessary to remain in the range [0, 1]. Note that because parochialism modifies social learning, the strength of selection on parochialism will be stronger when individuals rely on social learning more heavily.

For simulations in which multiple social learning strategies are possible (e.g. conformist or success-biased learning), the social learning strategy can also evolve. In these cases the offspring inherits the social learning strategy of its parent with probability 
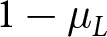
 and otherwise adopts a learning strategy at random from the set of allowable strategies.

#### 2.2.2 Model choice

Each individual considers 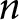 other agents (targets) from the previous generation to learn from, 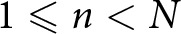. These targets will initially be chosen at random from the entire parent generation. The focal agent then evaluates each target, excluding a target with probability 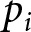 if it does not share its own marker. The agent then uses its learning strategy (see below) to learn from the remaining targets and/or via individual trial-and-error learning. It is possible for a highly parochial agent to have zero targets left after excluding outgroup targets. In this case, the agent will rely exclusively on individual learning.

#### 2.2.3 Learning

Each focal agent uses their learning strategy, 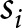, to acquire a trait value from their set of targets. There are three possible options:

- Unbiased transmission (UT). Choose a target at random from those under consideration and acquire their trait value.
- Conformist transmission (CT). Acquire the median trait value among the targets.
- Pay-off-biased transmission (PT). Acquire the trait value from the target with the highest pay-off. If two or more targets have the same high pay-off, choose one at random.

In our first analyses, we will exclusively consider agents using only UT. In later analyses, we will consider CT and PT separately and in tandem, always in competition with UT, and agents always use UT at the start of every simulation. We denote the trait value acquired by agent 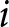 via social learning as 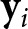.

Each agent 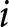 also acquires via individual trial-and-error learning a trait value 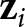, such that
(2)
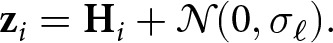


This individually learned trait value is a point in Cartesian space drawn from a bivariate normal distribution centred on 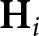 and a normalized standard deviation of 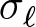, which represents the uncertainty or error associated with individual learning. The trait the agent ultimately adopts is an average of these individually and socially learned values, weighted by their reliance on social learning:
(3)
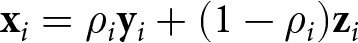


For simplicity, we model learning as a one-time event that determines the agent’s trait value and therefore their lifetime fitness, in line with most other models of social learning evolution. Scenarios in which iterated individual learning increases accuracy (Turner et al., 2023; West-Eberhard, 2003) can be approximated here by assuming smaller values for 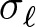.

After the learning stage, the agents in the previous generation are no longer relevant to the model dynamics and so they are removed from the simulation in a manner not entirely dissimilar from the 1976 film *Logan’s Run*.

#### 2.2.4 Pay-offs

Following Boyd and Richerson (1985), we use a Gaussian fitness function such that the maximum fitness for an agent in group 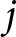 is 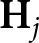, and their fitness declines with their trait’s Euclidean distance from that value. Written out, the pay-off 
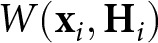
 to an agent 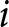 is given by the following:
(4)
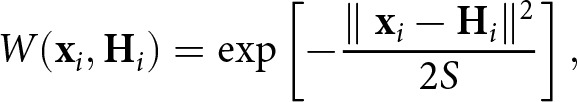


where the double-vertical lines represent the Euclidean distance between the two points, and the denominator 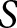 is a parameter controlling the intensity of selection (see Figure 1B).

To ensure reproducibility, the model was duplicate coded by PES in NetLogo 6.3 and by APV in Julia using the Agents.jl library (Vahdati, 2019). All results reflect the Julia version, for which code as well as simulation data are available at https://github.com/datadreamscorp/SimilarityBiasedLearning.

### 2.3 Outcome measures

The most important outcome measures are the average values of social learning reliance (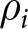) and parochialism (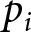) in each group. We also track the average group and population pay-offs and the frequency of each learning strategy (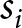) for each group. Each simulation was run for 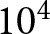 time steps, which was determined by visual inspection to be well above the typical time needed for these outcome measures to plateau; outcome measures reflect the state of the model at the conclusion of each simulation. The data we present are aggregated from 100 simulation runs for each parameter combination. All the results below use 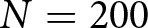 and 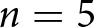. In the SI Appendix, we also present results for 
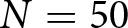
 and 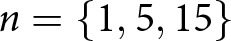 and show that they are qualitatively similar.

## 3. Results

In order to present our results in a way that maximizes understanding, we will begin with a baseline model that recovers the well-known result that social learning evolves more readily when individual learning is more error-prone. We will then show that when populations are sufficiently diverse in terms of the trait values individuals find optimal, higher probabilities of copying someone with a very different optimal trait value will decrease selection on social learning. Next, we will show that adding the ability to selectively filter out information from those who do not share social markers allows social learning to remain adaptive even in diverse populations, although this effect is strongest when markers are reliable indicators of adaptive trait values. Finally, we will show that our results regarding parochialism do not rely on unbiased social learning, but also hold when individuals can also take advantage of more cognitively advanced strategies like conformist or pay-off-biased transmission.

### 3.1 Social learning evolves when individual learning is more uncertain

**Figure 2.**
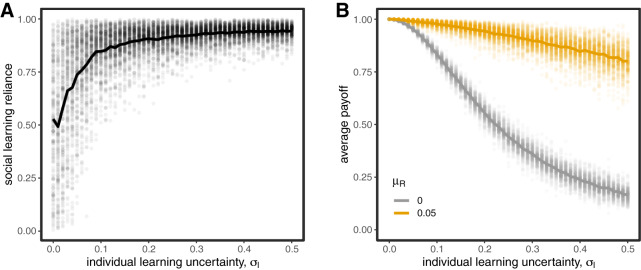
(A) Selection on social learning increases with extent to which individual learning is error-prone. (B) Pay-offs universally decrease when individual learning is more error-prone, but are substantially higher when social learning is allowed to evolve (
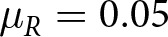
) than when agents must rely only on individual learning (
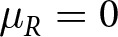
). Circles represent population means from individual simulation runs, with the solid lines connecting the means across runs.

We first ran simulations in which all individuals have the same adaptive trait value (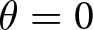) and are therefore effectively all members of the same group. These simulations did not allow for parochialism (
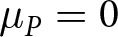
, 
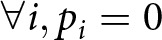
) and all social learning was unbiased (
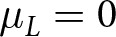
, 
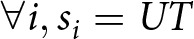
). When individual learning was very accurate (
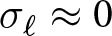
), social learning could still evolve by drift because successful individual learners made consistently good targets for social learners, who themselves then became good targets for social learning. Because selection on social learning was neutral, reliance on social learning took on the full range of values in 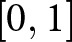. However, as individual learning became more error-prone, selection increasingly favored individuals that could rely on social learning, pushing values of 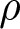 close to 1 (Figure 2A). We demonstrate that social learning here makes a tangible difference: although pay-offs universally decrease when individual learning is more error-prone, they are nevertheless substantially higher when social learning is allowed to evolve (Figure 2B).

### 3.2 Unbiased social learning does poorly in more diverse populations

**Figure 3.**
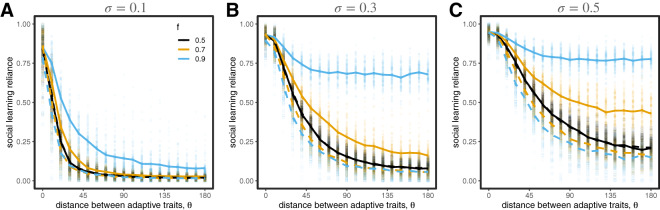
Social learning reliance becomes increasingly selected against as the distance between adaptive traits, 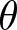, increases. This effect is marginally stronger when individual learning is less error-prone (when 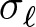 is smaller). The effect is also weaker when individuals belong to a majority group and stronger when they are in the minority – for each coloured pair of lines, the solid line is the majority (a proportion 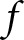 of the population) and the dashed line is the minority (a proportion 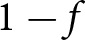). Circle and square markers represent means from individual simulation runs, with the solid lines connecting the means across runs.

The efficacy of social learning is highest when the traits that work best for target individuals are also effective for those who learn from those targets. Large-scale environmental change and spatial migration can disrupt these correlations, reducing the efficacy of social learning (Aoki & Feldman, 2014; Boyd & Richerson, 1985; McElreath et al., 2013; Turner et al., 2023). Here we show that intrapopulation diversity can have a similar effect. Recall that there are two groups of individuals within the population. As the maximally adaptive trait values for each group diverge (as 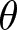 increases), unbiased social learning is increasingly ineffective and therefore selected against (Figure 3). This effect is stronger when individual learning is less error-prone (when 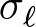 is smaller), because individual learning provides a more reasonable alternative to noisy social learning. The effect is also weaker when individuals belong to a majority group and stronger when they are in the minority, and this difference is amplified when the difference in relative size between the two groups is larger (when 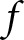 is larger). This is because members of the majority are more likely to choose a target that belongs to their own group purely by chance, whereas members of the minority are unlikely to find the behaviours adopted by the majority particularly adaptive (Figure 3). Of course, this result assumes, along with most prior models of social learning, that individuals cannot readily distinguish between others who are more or less likely to share adaptive behaviours.

### 3.3 Parochialism allows social learning to be adaptive in diverse populations

**Figure 4.**
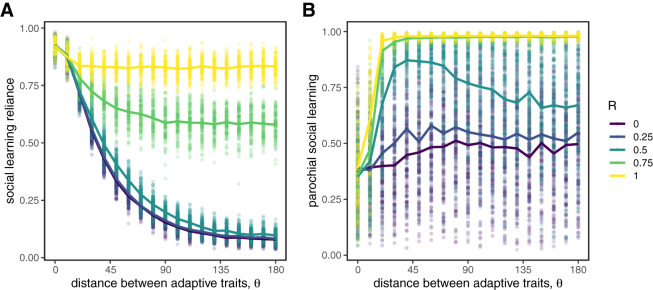
The evolution of parochial social learning. Social learning reliance (A) is maintained even for high values of 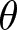 when parochialism is allowed to evolve, especially when markers are reliable indicators of group-linked adaptive trait values (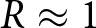). The evolution of high values of social learning reliance is always accompanied by parochialism (B), which otherwise can remain present via drift. Circles represent means from individual simulation runs, with the solid lines connecting the means across runs.

We now consider what happens to the evolution of social learning when individuals can use social markers to filter out targets who don’t share their markers. If markers are uncorrelated with group identity, there is, predictably, no effect. However, when the markers *do* convey reliable information about whether a target is likely to share a learner’s adaptive trait value, parochialism evolves and the evolution of social learning reliance is recovered (Figure 4A). The evolution of high values of social learning reliance is always accompanied by parochialism (Figure 4B), which otherwise can remain present via drift but will have no effect if individuals are not reliant on social learning. In SI Appendix Figure S1 we show this result is robust to changes in both population size and the number of targets considered by social learners.

**Figure 5.**
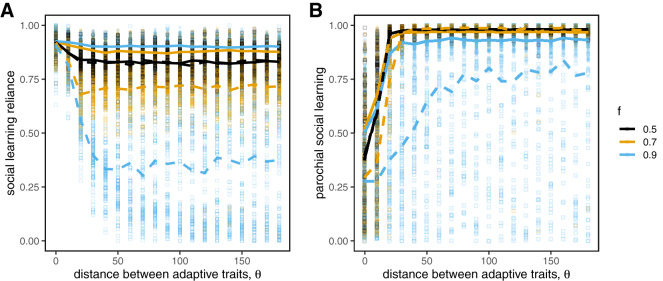
The evolution of parochial social learning among majority and minority groups when markers are maximally informative (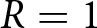). For each coloured pair of lines, the solid line is the majority (a proportion 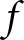 of the population) and the dashed line is the minority (a proportion 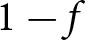). Circle and square markers represent means from individual simulation runs, with the solid lines connecting the means across runs.

The trend also holds when groups vary in size, although parochialism affects majority and minority groups differently (Figure 5). Majority groups are better able to take advantage of social learning and evolve strong parochialism to avoid accidentally learning from the minority. Minority groups don’t get as much benefit from parochialism; it’s often the case that the majority of their initial set of models are outgroup agents, and so we observe less reliance on social learning in the minority group. The strength of this effect is smaller but still present if more targets are initially observed (see Figure S1). In Figure S2, we also consider how relative group size interacts with the correlation between identity marker and adaptive trait, 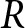. We find that minority groups are more strongly affected by decreasing correlations between marker and adaptive trait, reliably evolving less parochialism and less reliance on social learning overall. Majority groups are better able to capitalize on social learning even in the absence of markers, because randomly selected targets are more likely to share adaptive trait values.

It is worth noting that this result – that parochialism works less well for minority groups – only holds to the extent that minority individuals cannot use other means to learn directly from members of their ingroup. In cases where minority members can easily find ingroup targets for social learning, as when there is strong homophily for social interactions, this result will not apply. That said, in domains in which minority individuals do not have strong representation, they may struggle with trying to copy the techniques of the majority and be forced to rely more on noisy individual learning.@@@

### 3.4 Parochial social learning works similarly when combined with conformist and pay-off-biased transmission

Our results above rely on the assumption that social learning is unbiased; that is, individuals choose their targets at random. Other social learning strategies like conformist and pay-off-biased transmission have been suggested to reduce noise and improve the efficacy of transmission (Boyd & Richerson, 1985; Henrich & Boyd, 1998; Kendal et al., 2018; McElreath et al., 2008, 2013; Muthukrishna et al., 2016; Whiten, 2019). Here we investigate whether these strategies provide similar benefits in diverse societies, and whether parochialism provides any additional benefit. We set 
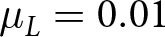
 and allowed either conformist transmission, pay-off-biased transmission, or both to evolve. We will show that our results are robust to the presence of both of these learning strategies; however, we also show that the ability to evolve pay-off-biased social learning can create traps for the more slowly evolving group at suboptimal fitness valleys.

#### 3.4.1 Conformist transmission

**Figure 6.**
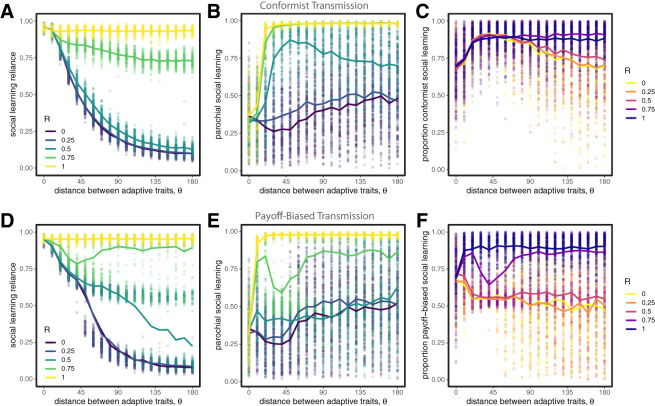
Evolution of traits when agents can evolve either conformist transmission (top row) or pay-off-biased transmission (bottom row). The evolution of high values of social learning reliance is always accompanied by parochialism; compare (A,B,D,E) to Figure 4. Conformist transmission is favoured in almost all cases (C), whereas pay-off-biased transmission is only favoured when group markers are informative of adaptive traits (i.e. when 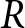 is high; F). Circles represent means from individual simulation runs, with the solid lines connecting the means across runs.

Our results are qualitatively the same when conformist transmission can evolve in competition with unbiased social learning (Figure 6A,B). Conformist transmission allows agents to quickly reduce the noise in social information, and therefore has an advantage over unbiased transmission, but when populations are diverse this advantage can only be achieved when paired with parochialism. We find that conformist transmission reliably evolves in most cases, although selection is not as strong in cases when 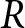 is low and 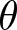 is high (Figure 6C). We also find that conformity evolves even with very low reliance on social learning when group markers are unreliable indicators of adaptive trait values (when 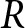 is low). This is because *any* social learning will tend to benefit from conformity, which minimizes the influence of observing non-adaptive behaviours as long as they are relatively rare. If groups are of unequal size (e.g. 
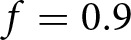
), our simulations indicate that conformist transmission works well for those in the majority group, because most sampled individuals will be in the majority, whereas for those in the minority conformist transmission is under neutral selection with unbiased learning, both of which are highly error prone. The only exception is when groups share the same adaptive trait values (i.e. 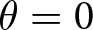). In this case, conformity is favoured for everyone.

#### 3.4.2 Pay-off-biased transmission

Our results are also qualitatively very similar when we allow for pay-off-biased transmission to compete with unbiased transmission (Figure 6D,E). Pay-off-biased transmission allows agents to selectively learn from successful targets and therefore reduced the chance that they will copy someone with low-quality information, but in diverse societies we again find that this advantage can only be achieved when paired with parochialism.

The opportunity to evolve pay-off-biased transmission can also lead to a particularly interesting outcome not seen with other learning strategies. Specifically, the amount of reliance on social learning that evolves appears to have multiple equilibria when 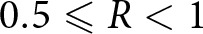 (Figure 6D). These outcomes reflect cases in which one group evolves pay-off-biased social learning and the other group does not. The reason this occurs is due to path-dependent coevolutionary dynamics among pay-off-biased transmission, parochialism, and social learning reliance. In diverse populations (when 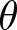 is large), social learning is only effective if agents have evolved parochialism (with the exception of cases where one group constitutes the overwhelming majority of the population). At 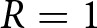, group markers are very reliable indicators of adaptive trait value, and the benefit of parochial social learning is unambiguous. On the other hand, when 
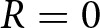
, parochialism provides no benefit. In the middle, at intermediate values of 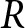, parochial social learning can still offer a benefit over individual learning, but the advantage must overcome the noise in the markers’ signals. Here is where the path dependency comes in.

What can happen is that one group evolves pay-off-biased parochial social learning and its group members rapidly converge to their adaptive maximum. For the second group to perform similarly, they must evolve strong parochialism *before* evolving pay-off-biased social learning. Otherwise, non-parochial pay-off-biased social learning dooms them, because those in the population with the highest overall pay-offs will tend to be from the outgroup. And because pay-off-biased transmission can evolve by neutral drift when social learning reliance is low, populations can become trapped in scenarios where it is better to use noisy individual learning than to learn socially from high-performing individuals whose behaviours do not yield the same benefits to learners from other groups. Interestingly, in this asymmetric scenario, the high-performing group often *loses* parochialism after some time and relies solely on pay-off-biased transmission, because its members do consistently better than members of the other group, and thus pay-off becomes a better proxy of adaptive trait than (not fully reliable) markers. If the second group does eventually adopt parochial social learning, its pay-off can rapidly increase, which leads to a catastrophic collapse of social learning in the first group if they have abandoned parochialism, as pay-off information loses its value as a group marker. The two groups then continue to stochastically cycle through periods high and low reliance on social learning, each going through opposing periods of high and low pay-off. This result occurs only for intermediate values of 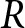, and only when groups fail to evolve parochial pay-off-biased learning at the same time, but it is not an implausible dynamic for how groups can gain and lose dominance over time. We illustrate these dynamics, contrasting them with more typical outcomes, in Figure S3.

#### 3.4.3 Competition among all three social learning biases

**Figure 7.**
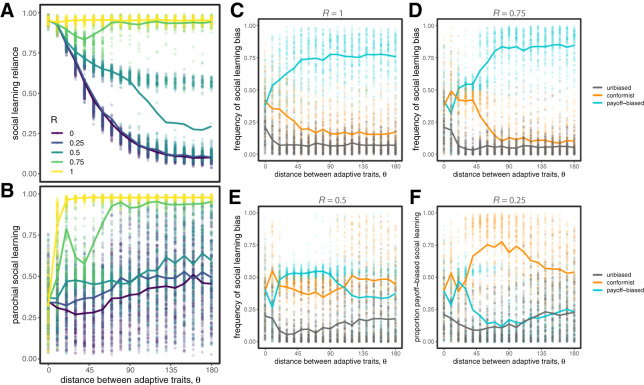
Evolution of traits when all three social learning biases (unbiased transmission, conformist transmission, and pay-off-biased transmission) compete. The evolution of social learning reliance (A) and parochialism (B) are similar to previous results. A closer look at competition between learning biases show that whether conformist or pay-off-biased transmission is favoured depends on 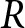, the extent to which group markers are informative of adaptive traits (C–F). Results for 
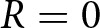
 are qualitatively indistinguishable from those for 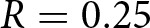 and are therefore not shown. Circles represent means from individual simulation runs, with the solid lines connecting the means across runs.

Finally, we considered scenarios in which unbiased, conformist, and pay-off-biased transmission could all compete. The qualitative nature of our results remain the same, with parochialism stabilizing social learning reliance in diverse populations (Figure 7A,B). When group markers are reliable indicators of adaptive trait values (when 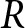 is large), pay-off bias dominates, because it is more immediately effective as a noise-reducing learning strategy than either unbiased or conformist transmission. As 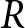 gets smaller, pay-off-biased transmission performs much worse, because agents can’t use identity information to determine which targets are using behaviours that are right for them (Figure 7C–F). In these cases, conformist transmission works best, because it allows learners to avoid the catastrophic choices; however, in diverse populations (when 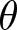 is large), it still isn’t as good as relying solely on individual learning.

## 4. Discussion

Parochial (or similarity-biased) social learning is adaptive in diverse populations if it directs attention to targets most likely to be performing behaviours that will be useful to the learner. The cost of ignoring information from outgroup individuals is outweighed by the signal boost parochialism can provide. This is true even if identity markers are not perfect indicators of aligned circumstances, and even when social learning can be otherwise bolstered by conformist or success-biased transmission. These results help explain why we see such widespread preferences for learning from or otherwise engaging with individuals or media marked in ways that indicate a shared identity.

These results contribute to theories of cultural evolution in several ways. First, they help to justify prior theoretical work that has relied on the assumption that individuals would prefer to learn from similarly marked targets (Akerlof & Kranton, 2000; Boyd & Richerson, 1987; Bunce, 2021; McElreath et al., 2003; Smaldino & Turner, 2022). Second, they provide qualitatively testable hypotheses regarding the presence or absence of biases for learning from similar others, which should depend on the difficulty of individual learning, the diversity of possible adaptive behaviours available to learners, and the extent to which identity markers correlate with those adaptive behaviours. Third, they strengthen the theoretical foundation for cultural group selection, which can operate when transmission-isolating mechanisms maintain between-group differences in behaviours, norms, and institutions (Durham, 1991; Henrich, 2004; Richerson et al., 2016). A bias for learning preferentially from similar targets is just such a mechanism, enhancing the ability of group-level selection to produce and stabilize prosocial or otherwise group-beneficial norms and institutions among cultural groups.

Our results may also help to explain a widely acknowledged phenomenon in the education literature concerning the benefits experienced by students when learning from teachers with shared identities, usually focusing on race but also including gender or sexual orientation (Dee, 2005; Gershenson et al., 2022). Our analysis implies that this benefit, when present, may not stem intrinsically from shared identities per se, but from the extent to which the markers of identity signal incentives, norms, and circumstances are shared between teachers and students. In cases where those identity markers fail to accurately signal such associations, shared identity between teachers and students may not yield a benefit. Conversely, teachers whose overt identities differ from their students’ in terms of ethnicity, gender, or sexual orientation but align on other meaningful dimensions may nevertheless be preferred, depending on the circumstances and interests of the students and available teachers.

More generally, all communication involves manipulation insofar as its function for the sender is to alter the behaviour or motivations of the receiver (Owings & Morton, 1997). When interests are not aligned, senders may be incentivized to provide untrue or misleading information (Crawford & Sobel, 1982). To the extent that shared identities are indicative of aligned interests or goals, the presence of incentives for deception are likely to further strengthen selection for parochial social learning. Kline (2015) notes that teaching can be viewed as a form of altruistic cooperation, because the teacher incurs a cost to provide a benefit to the learner. Thus, ensuring that the interests of teachers and students are reasonably aligned will help ensure that teachers are providing instruction that is both veridical and beneficial to the student.

In our model we downplayed the normative and frequency-dependent properties of many behaviours. The benefit of a behaviour may depend on how many others also perform it. This is captured somewhat by our assumption that different groups benefit differentially from the same behaviour, which could represent a tendency for a behaviour to involve coordination with a homophilous ingroup. But coordinative behaviours are often more nuanced than this. In particular, non-normative behaviours may be punished despite having the potential to make a group better off if widely adopted (Boyd & Richerson, 2002; Pisor & Ross, 2024; Smaldino & O’Connor, 2022). If learning by direct feedback from miscoordination is too slow or costly, selection on parochialism will again be further strengthened (Saunders, 2023).

Our model structure is inspired by prior models of the evolution of social learning in spatially varying environments, in which different spatial locations have different locally adaptive traits. Such models have shown that when migration between patches increases, selection on unbiased social learning is weakened (Aoki & Feldman, 2014; Boyd & Richerson, 1985; Henrich & Boyd, 1998; McElreath et al., 2013). Overcoming this limitation has been a key focus of the literature on conformist- or consensus-biased social learning. By expressing a bias toward more commonly observed behaviours, conformity down-weights information from targets who likely learned their behaviours in other environments, as long as they are in the minority. However, if local learners are in the minority, conformist learning can fail. Moreover, prior work on conformist social learning typically assumes that behaviours used by local majorities will be similarly adaptive for everyone. This assumption fails to account for the very different microenvironments – social networks, norms, socioeconomic opportunities, etc. – that members of different groups may face even when living in the same geographical areas. Parochial social learning solves this problem by directing the attention of learners toward members of their own group.

Our analysis indicates that parochialism has less-noticeable benefits for members of small minority groups. This result relies on two assumptions: that informational signals from minority group members are likely to be noisier due to their smaller relative population size, and that because of their low representation in the general population, excluding outgroup targets from social learning may lead to an exclusion of *all* targets, forcing a reliance on individual learning. In such cases, minority individuals will, as a consequence, have lower average pay-offs compared to members of the majority. We view this scenario as plausible under a wide range of conditions. It is a well-known result in demography that members of minority groups encounter members of the majority more often than members of the majority encounter members of the minority (Richardson et al., 2021; Sigelman et al., 1996). That said, scenarios are likely common in which minority members have sufficient homophilous assortment such that parochialism is as adaptive – if not more so – for them as it is for members of the majority. The applicability of our findings to minority–majority interactions depends in part on the strength of such assortment.

We focused on two main sources of uncertainty in the acquisition of adaptive behaviour: noisy individual learning and a diverse set of adaptive trait values among individuals. There are other sources of potential uncertainty we did not explore but that may contribute to the evolution of social learning, including temporal variation, number of learning opportunities, and the range of possible behaviours (Turner et al., 2023). Perhaps most notably, we held the state of the environment constant over time, so that the adaptive trait values are fixed for each subpopulation. The world often does change between and even within generations, from environmental, technological, and cultural change, and modelling has indicated that this type of variability can matter in distinct ways (Aoki & Feldman, 2014; McElreath et al., 2013). Our understanding of parochial social learning must ultimately include its response to temporally varying environments. We chose to ignore this source of variation in the present study for two reasons. First, many of the results for temporally varying environments are similar or identical to results for spatially varying environments, which are the basis for the model we study here, so many of our results will likely hold for a temporally varying environment as long as the environment does not change so often as to render any intergenerational social learning useless (Boyd & Richerson, 1985; McElreath et al., 2013; Turner et al., 2023). Pragmatically, the introduction of temporally varying environments also creates multiple new degrees of freedom for our model. Because we considered at least two simultaneous adaptive trait values (i.e. the maximally adaptive behaviours for each of two subpopulations), we had to track not only how a single trait value varies over time, but how the relationship *between* the traits varies. Moreover, a sudden change in adaptive trait value carries difficulties for agent-based models of discrete populations. Because we are explicitly simulating how a population evolves over time, catastrophic events require substantially longer evolutionary time to achieve steady-state equilibrium learning strategies. Given the complexity of our current model and the richness of our results, we have chosen to put off an analysis of temporally varying environments for future exploration.

We found that ability to evolve pay-off-biased social learning could work against disadvantaged groups (i.e. those slower to adopt high-pay-off behaviours). In the absence of pre-existing parochialism, a bias for learning from successful individuals caused these individuals to consistently copy members of the better-performing group, leading to maladaptive outcomes. In such cases, noisy individual learning performed better than pay-off-biased social learning, leaving the group at a quasi-permanent disadvantage. When social markers were highly reliable indicators of adaptive trait values, parochialism could readily evolve and help these groups overcome their disadvantage, because even small amounts of parochialism were adaptive. But when correlations between markers and adaptive traits were positive but imperfect, higher levels of parochialism were required to cut through the noise, leading to a fitness valley for any social learning that evolved under strong pay-off bias but low parochialism. This dynamic may help to explain some intergroup differences, particularly when correlations between group identities and successful behavioural strategies are present but noisy.

We would be remiss if we failed to discuss the downsides of relying on visibly marked social categories for social learning and related decision-making. Defining people in terms of social categories can quickly lead to harmful stereotypes and dehumanization of people seen as ‘other’, the consequences of which vary from moderate inconvenience to systemic inequality to extreme violence. Parochialism is also likely to facilitate the spread of misinformation and an exacerbation of polarization. The phenomenon of affective polarization, for example, is widespread in the United States, whereby partisans may make political decisions based less on policy preferences than on an animosity to anything desired by the opposition party (Iyengar et al., 2019). The loss of the fairness doctrine in the United States – which required broadcasters to devote some of their airtime to discussing controversial matters of public interest, including contrasting views on those matters – has contributed to the now-widespread media strategy of marking information as identity-linked (Simmons, 2022). When combined with parochial social learning, marking information with identity tags is likely to promote the creation of subpopulations with increasingly little overlap in media consumption, contributing to polarization (Klein, 2020; Taibbi, 2019) as well as the potential for entrenched social immobility (Bowles & Gintis, 2004). Parochial social learning has also been studied as ‘outgroup aversion’, which is the tendency to avoid adopting behaviours associated with an outgroup. When widespread adoption across group boundaries is societally beneficial, as in the case of public health measures during a global pandemic, the results can be catastrophic (Jones & McDermott, 2022; Smaldino & Jones, 2021).

Indeed, being overly parochial is likely at odds with a cohesive, pluralistic society (Mounk, 2023), at least when identity distinctions are those that drive groups apart. Although our analysis focused on cases in which the best strategies for each group yielded equal pay-offs, this will not always be the case due to structural inequalities. Moreover, the circumstances that lead to differential adaptive strategies for different groups are unlikely to be constant over time, but are at least partially a function of social organization. Thus, decreasing the adaptive nature of parochialism may benefit diverse societies in the long run. This requires the creation of an equitable society in which, on average, individuals using the same strategies have largely similar outcomes.

For the sake of tractability, our model assumed that groups and social markers were discrete and fixed, implying that individuals have a single identity that is consistent throughout time and inherited across generations. In reality, people belong to multiple groups, identity is flexible and context-dependent, and identities may shift over time (Roccas & Brewer, 2002; Smaldino, 2019; Swann et al., 2012). For this reason, what constitutes an ingroup – and who counts as a member thereof – is often complicated. One can think of similarity-biased social learning as a heuristic. When people don’t know what to do or who to learn from, they can use social identity markers as indicators of shared norms, goals, and circumstances. Importantly, over the course of the lifespan (or even of a given week), people have to learn *lots* of things, and it seems unlikely that they develop a unique strategy for learning every behaviour in every context. Rather, theories of bounded rationality (Gigerenzer & Selten, 2002) imply that we should develop meta-strategies for learning that work well across scenarios (Bednar & Page, 2007). In this way, proclivities for similarity-biased social learning might serve us well most of the time, despite leading us astray some of the time. In other words, parochial social learning can still be adaptive despite leading to adverse consequences on occasion. On the other hand, such occasional missteps may also be catastrophic when they lead people to ignore information originating from an outgroup that is existentially imperative to act upon, as in the case of some public health or climate change interventions. If the future is to become increasingly uncertain, parochialism may appear increasingly attractive, and people may increasingly cling to insular communities, rejecting social information from outsiders. That would be unfortunate.

## Supporting information

Smaldino and Velilla supplementary material 1 Smaldino and Velilla supplementary material

Smaldino and Velilla supplementary material 2 Smaldino and Velilla supplementary material

Smaldino and Velilla supplementary material 3 Smaldino and Velilla supplementary material

Smaldino and Velilla supplementary material 4 Smaldino and Velilla supplementary material

## Supplementary material

The supplementary material for this article can be found at https://doi.org/10.1017/ehs.2024.46.

## Research transparency and reproducibility

Model code in Julia is available at https://github.com/datadreamscorp/SimilarityBiasedLearning. The data from our model simulations are available here at https://datadryad.org/stash/share/uHViPgb3xpC2vN5HEhdHbwjLJ4e8d0Th-bVzdmLIBw8.
