# Supplementary material for: The evolution of similarity-biased social learning
Source: Evol Hum Sci. 2025 Jan 20;7:e4. doi: 10.1017/ehs.2024.46 (PMC11859121; doi:10.1017/ehs.2024.46)
Supplement: Smaldino and Velilla supplementary material 1 — Smaldino and Velilla supplementary material [file S2513843X2400046Xsup001.pdf]

SUPPLEMENTARY MATERIAL: THE EVOLUTION OF SIMILARITY-BIASED SOCIAL LEARNING

PAUL E. SMALDINO[1,2,*] AND ALEJANDRO PÉREZ VELILLA[1]

In this document, we present supplementary figures for the study "The Evolution of Similarity-Biased Social Learning" to indicate the robustness of our results for a wider range of parameters than those explored in the main text and to illustrate some interesting dynamics. First, we reproduce the outcomes shown in main text Figure 4 for $N = 50, 200$ and $n = 1, 5, 15$ (Figure S1). Next, we reproduce the outcomes shown in main text Figure 5 for $R = 0, 0.25, 0.5, 0.75, 1$ (Figure S2). Finally, we we illustrate some of the model dynamics in which both parochialism and success-biased social learning can evolve, illustrating both typical dynamics, cycling, and the emergence of between-group differences (Figure S3).

[1]Department of Cognitive & Information Sciences, University of California, Merced
[2]Santa Fe Institute
E-mail address: paul.smaldino@gmail.com.

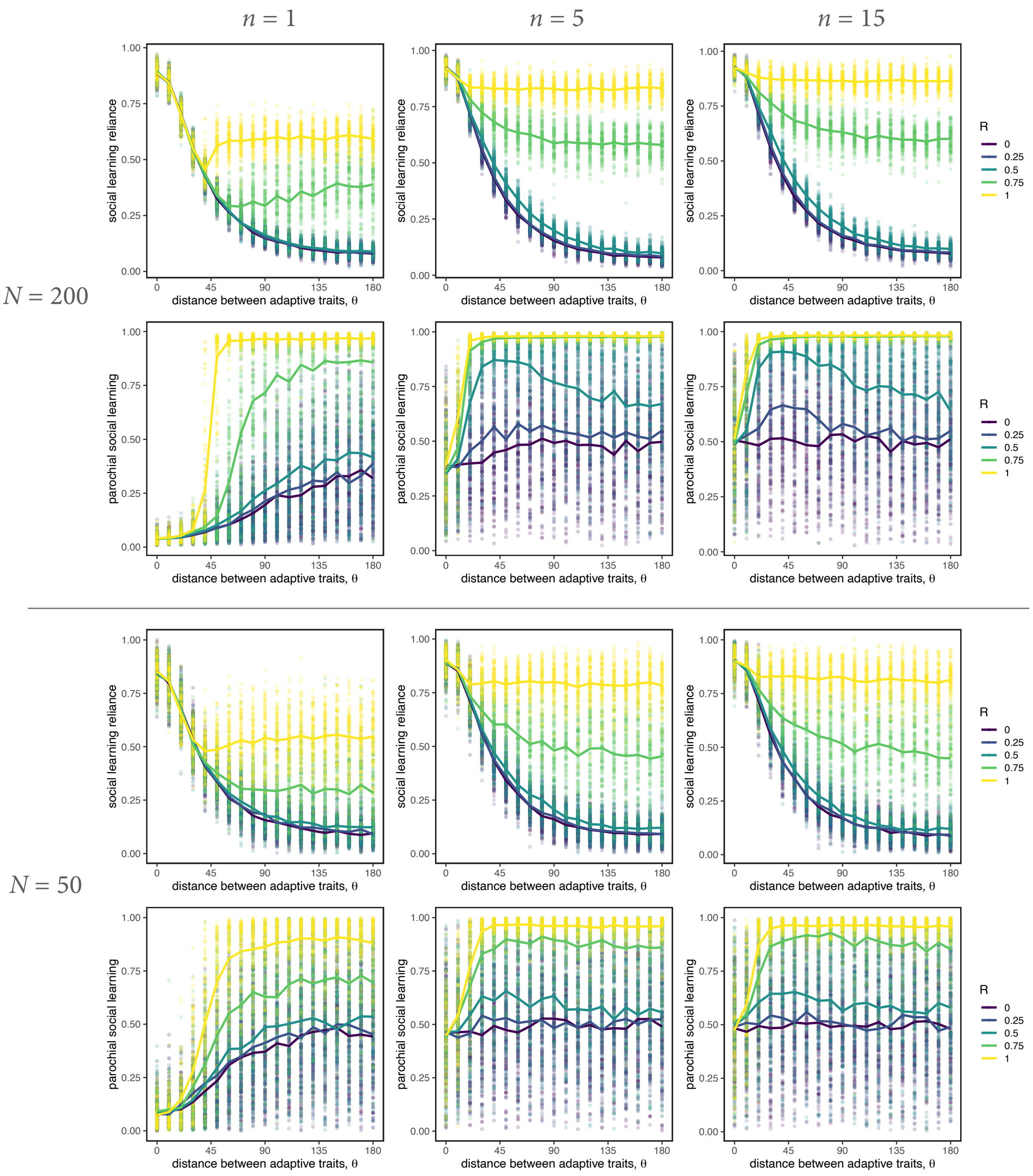

Figure S1. The evolution of parochial social learning for varying n, N, and R. For each value of N, top row: the evolution of social learning reliance as a function of θ; bottom row: the evolution of parochial social learning as a function of θ. All other parameters are as given in main text Table 1. Circles represent means from individual simulation runs, with the solid lines connecting the means across runs.

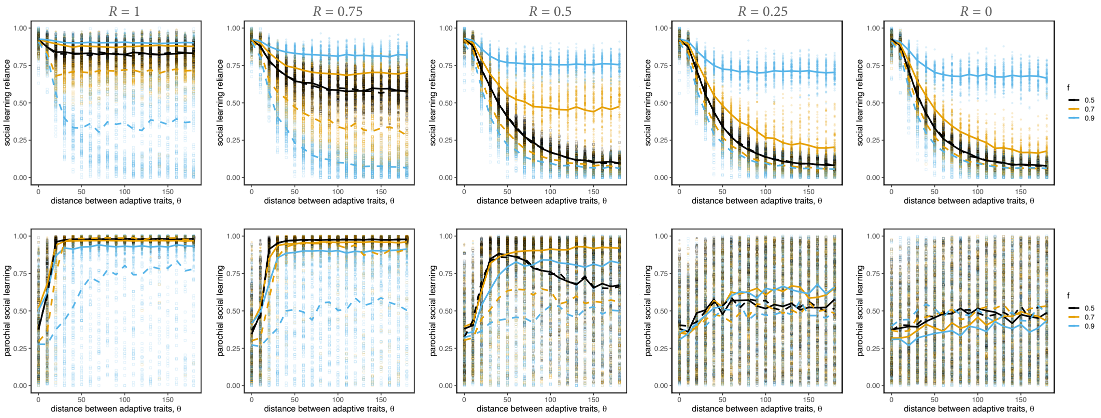

FIGURE S2. The evolution of parochial social learning among majority and minority groups for varying R. For each colored pair of lines, the solid line is the majority (a proportion f of the population) and the dashed line is the minority (a proportion $1-f$). All other parameters are as given in main text Table 1. Circle and square markers represent means from individual simulation runs, with the solid lines connecting the means across runs.

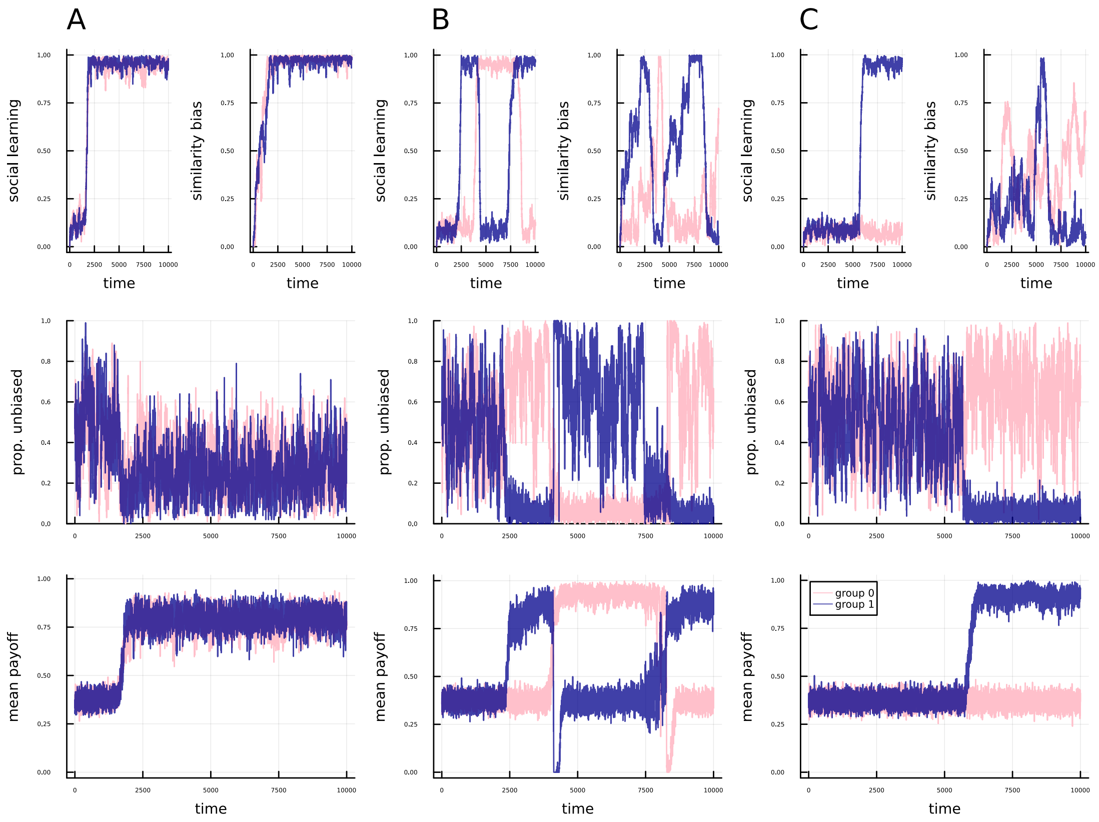

FIGURE S3. Distinct cases of group differences in evolution of parochialism and payoff-biased social learning under imperfect correlation between group tag and group membership. When tags are not perfect signals but still have a significant probability of holding useful information (here $R = 0.75$), both groups can either **(A)** achieve high payoff-biased social learning at the same time, or **(B)** enter a parochialism-fueled tug-of-war, in which at a given moment they exchange positions between being high-payoff, non-parochial payoff-biased social learners and being being low-payoff, low reliance on social learning. Interestingly, low-payoff groups can take the place of high-payoff groups by evolving highly parochial payoff bias and collapsing the payoff of high-payoff groups (prompting them to quickly swap to low social learning reliance), only to abandon parochialism once they become the advantaged group, giving the chance for the formerly high-payoff group to recover their position by evolving parochialism. **(C)** As group tags decrease in reliability (here $R = 0.5$), and given that groups don't evolve parochial learning at the same time, then the oscillations from (B) become a stable payoff hierarchy, as parochialism loses its strategic edge for the disadvantaged group. In these runs, $N = 200$, $\theta = 180°$, $f = 0.5$, $\sigma_l = 0.3$, $n = 5$, $s_i \in \{UT, PT\}$. The remaining parameters have the non-zero values specified in main text Table 1.
