# Supplementary material for: The evolution of similarity-biased social learning
Source: Evol Hum Sci. 2025 Jan 20;7:e4. doi: 10.1017/ehs.2024.46 (PMC11859121; doi:10.1017/ehs.2024.46)
Supplement: Smaldino and Velilla supplementary material 4 — Smaldino and Velilla supplementary material [file S2513843X2400046Xsup004.docx]

**The Evolution of Similarity-Biased Social Learning**

**Social Media Summary**

Why do we often prefer to learn from people who are similar to us and ignore people we view as different? We present a cultural evolutionary model that demonstrates how, in diverse populations, parochial social learning can be adaptive while other social learning strategies—like learning from successful individuals or averaging over many observations—can fail. We discuss implications for stereotypes and polarization.
